# Assessment of Probiotic Properties of *Lactobacillus salivarius* Isolated From Chickens as Feed Additives

**DOI:** 10.3389/fvets.2020.00415

**Published:** 2020-07-17

**Authors:** Jian Wang, Muhammad Ishfaq, Yuquan Guo, Chunli Chen, Jichang Li

**Affiliations:** ^1^College of Veterinary Medicine, Northeast Agricultural University, Harbin, China; ^2^Heilongjiang Key Laboratory for Animal Disease Control and Pharmaceutical Development, Harbin, China

**Keywords:** *Lactobacillus salivarius*, growth performance, *Escherichia coli*, heat stress, oxidative stress

## Abstract

The continued use of sub-therapeutic antibiotics as feed additives in the poultry industry improved health and growth performance. However, the resulting antibiotic resistance increasingly becomes a major threat to public health. Probiotics are promising alternatives for the antibiotics used in poultry industry. The aim of this study was to evaluate the probiotic properties of *Lactobacillus salivarius* as feed additive in chickens. White leghorn chickens were randomly assigned to experimental groups. Effects of *Lactobacillus salivarius* supplementation on growth performance, resistance to *Escherichia coli* O78 challenge and heat-stress, and immune response after vaccinated with attenuated infectious bursal disease virus (IBDV) vaccine were determined. The results showed that *Lactobacillus salivarius* supplementation improved growth performance, such as weight and longer shank length, increased relative weights of the immune organs and decreased concentrations of odor-causing compounds. In addition, *Lactobacillus salivarius* supplementation alleviated organ injury caused by *Escherichia coli* O78 challenge and heat stress. Furthermore, *Lactobacillus salivarius* results in enhanced immune response after IBDV vaccine immunization, enhanced specific antibody and IFN-γ production, and lymphocyte proliferation. Our results revealed a tremendous potential of *Lactobacillus salivarius* as antibiotics' substitute in poultry production.

## Introduction

Sub-therapeutic antibiotics are widely used as growth promoters in the poultry industry for decades (1). However, the continued use of antibiotics as feed additives causes direct selection and rapid spread for antibiotic-resistant strains (1, 2). The resulting antibiotic resistance not only complicate the therapy of bacterial infections of chickens, but also become a major threat to public health (2). The European Union has completely banned the use of sub-therapeutic antibiotics in feed industry (1). Although this approach has helped to curb the sharp rise of antibiotic resistance, it has also brought great challenges to the poultry industry, such as the reemergence of the disease (3, 4). Therefore, the development of safe alternatives to antibiotics has gained global focus in recent years.

Avian pathogenic *Escherichia coli* (APEC) O78 has been associated with colibacillosis in chickens, which is one of the most common diseases of chicken farms (5). Sub-therapeutic antibiotics used as feed additives were effective against APEC infection. However, due to pronounced increase in the emergence of multiple resistance of *Escherichia coli* infection, it is necessary to develop safe alternative to reduce *Escherichia coli*-infection (5). Heat stress is one of the most challenging environmental conditions for the poultry industry and has negative effects on health and productivity of chickens (6). It has been demonstrated earlier that multiple vital organs showed different degrees of damage along with oxidative stress when chickens were exposed to heat stress (7). Infectious bursal disease virus (IBDV) infection can cause huge economic loss to poultry production, vaccination is one of the important approaches to prevent this disease (8). However, due to immunosuppression, the efficacy of vaccination is often unexpected because of poor response to IBDV vaccine (8). Oxidative stress is a common reason for the immunosuppression in poultry (8, 9). Hence, nutritional feed additives to minimize the effects of oxidative stress have gained an increasing focus in recent years.

*Lactobacillus*, as the first officially approved microbial feed additives, have incomparable advantages due to their low production cost and high availability (2). Due to the beneficial effects on growth performance, immunity and antioxidant capacity, *Lactobacillus* have been used in feed industry for decades (2). For example, chickens fed with *Lactobacillus plantarum* LP-8 increased the content of intestinal mucosal immune globulin A and enhanced the antioxidant capacity (10); Oral administration of a probiotic mixture, including *Lactobacillus reuteri*, effectively ameliorated intestinal injury of chickens that subjected to heat stress (11). Compared to the proverbial probiotics, such as *Lactobacillus reuteri, Lactobacillus plantarum* and *Lactobacillus casei*, the health-beneficial effects of *Lactobacillus salivarius* are inadequately researched in chickens. This study confirmed that *Lactobacillus salivarius* feeding improved growth performance, reduced the negative effects caused by *Escherichia coli* O78 and heat stress, and enhanced immune response after vaccination.

## Materials and Methods

### Chickens

All animal experiments were conducted under the approval of Laboratory Animal Ethics Committee of Northeast Agricultural University (Heilongjiang, China) in accordance with Laboratory Animal-Guideline for ethical review of animal welfare (GB/T35892-2018, National Standards of the People's Republic of China). 1-day-old white leghorn chickens were purchased from a local commercial hatchery (YiNong, Harbin, Heilongjiang, China). The animals were housed in wired cages (100 cm long × 80 cm wide × 50 cm high/cage). Room temperature was set to 32 ± 2°C for the first week and gradually reduced to 25 ± 2°C. Water was provided *ad libitum* and fed basal diet, mainly consist of corn and soybean meal, provided by Heilongjiang Huifeng Biotechnology (Heilongjiang, China). The chickens were confirmed negative to *Mycoplasma gallisepticum, Mycoplasma synovium, Salmonella pullorum*, and *Escherichia coli* O78, and did not undergo vaccination.

### Study Design

Study 1: A total of 480 1-day-old chickens were randomly assigned to eight experimental groups (*n* = 60). Chickens in control group were fed basal diet; chickens in other experimental groups were fed basal diet supplemented with *Lactobacillus salivarius* of 10^7^, 10^8^, and 10^9^ CFU/kg of feed, respectively. Samples including feces, spleen, bursa and thymus tissues were collected at 4 and 8 weeks of age for further analysis. The study 1 was designed to evaluate the effects of dietary *Lactobacillus salivarius* supplementation on growth performance, immune organ index, and fecal major odor-causing compounds concentrations of chickens.

Study 2: To evaluate the effect of *Lactobacillus salivarius* supplementation on *Escherichia coli* O78-related mortality, a total of 400 1-day-old chickens were randomly assigned to four experimental groups (*n* = 100). Chickens in control group were fed basal diet; chickens in *Lactobacillus salivarius* group were fed basal diet supplemented with *Lactobacillus salivarius* of 10^8^ CFU/kg of feed. Indicated group were orally administered with 10^6^ CFU *Escherichia coli* O78 (CVCC1553, China veterinary culture collection center) at day 7. The chickens that survived at 8 weeks of age were counted (the moribund animals euthanized by cervical dislocation and recorded as mortality). The above experiments performed three times and the death rate was calculated.

To further elucidate the protective effects of *Lactobacillus salivarius* supplementation against *Escherichia coli* O78, a total of 200 1-day-old chickens were randomly assigned to four experimental groups (*n* = 50), control group and *Lactobacillus salivarius* group treated same as stated above. Samples including feces, ileal content and ileum tissues were collected 7 days post-*Escherichia coli* O78 challenge.

Study 3: A total of 160 1-day-old chickens were randomly assigned to eight experimental groups (*n* = 20). Chickens in control group were fed basal diet; chickens in *Lactobacillus salivarius* group were fed basal diet supplemented with *Lactobacillus salivarius* of 10^8^ CFU/kg of feed. 21-days-old chickens in indicated group were moved to a preheated air chamber (Suzhou Fengshi Laboratory Animal Equipment Co. Ltd, China) at 42 ± 2°C. Samples including heart tissues and serum were collected after 0, 1, 3, 5, and 10 h durations of heat stress. The study 3 was designed to evaluate the effects of dietary *Lactobacillus salivarius* supplementation on the cardiac response of chickens to acute heat stress.

Study 4: A total of 160 1-day-old chickens were randomly assigned to eight experimental groups (*n* = 20). Chickens in control_NT and control_HT group were fed basal diet; Chickens in *Lactobacillus salivarius*_NT and *Lactobacillus salivarius*_HT group were fed basal diet supplemented with *Lactobacillus salivarius* of 10^8^ CFU/kg of feed. 21-days-old chickens in control_HT and *Lactobacillus salivarius*_HT group were exposed to heat stress (37 ± 2°C) for 3 h once a day for 15 days; 21-days-old chickens in control_NT and *Lactobacillus salivarius*_NT group were given room temperature (25 ± 2°C), samples including serum and ileum tissues were collected at 7 and 14 days post-heat stress. The study 4 was designed to evaluate the effects of dietary *Lactobacillus salivarius* supplementation on the response of chickens to circular heat stress.

Study 5: A total of 120 1-day-old chickens were randomly assigned to eight experimental groups (*n* = 15). Chickens in control group were fed basal diet; chickens in control + cyclophosphamide (cy, Pude Pharmaceutical, Shanghai, China) group were fed basal diet and intramuscular injection of cy at 50 mg/kg body weight once a day at age of 8–10 days; chickens in *Lactobacillus salivarius* group were fed basal diet supplemented with *Lactobacillus salivarius* of 10^8^ CFU/kg of feed; *Lactobacillus salivarius* + cy group were fed basal diet supplemented with *Lactobacillus salivarius* of 10^8^ CFU/kg of feed and intramuscular injection of cy at 50 mg/kg body weight once a day at age of 8–10 days. All chickens were vaccinated with an attenuated IBDV vaccine (Strain B87, Zhejiang EBVAC Bioengineering, Hangzhou, China) at day 14. Samples including serum and spleen tissues were collected 7- and 14-days post-immunization. The study 5 was designed to evaluate the effects of dietary *Lactobacillus salivarius* supplementation on immune response of chickens.

### *Lactobacillus salivarius* Isolation and Probiotic Potency Assay

*Lactobacillus salivarius* was isolated from feces of control group chickens (21-days-old). One grams feces was added to 10 mL sterile PBS and mixed fully, then 200 μL supernatant spread on MRS agar (Hopebiol, Qingdao, China) and aerobic culture at 37°C for 18-24 h. Suspected colonies were collected and grown in MRS liquid medium (Hopebiol, Qingdao, China). Bacterial DNA was extracted using MagPure Stool DNA KF kit (Magen, China) following the manufacturer's instructions. PCR amplification using 16S rRNA common primers, primer sequence as 27F: 5′-AGAGTTTGATCCTGGCTCAG-3′; 1492R: 5′-GGTTTACCTT GTTACGACT T-3′. The PCR reaction condition is 95°C 5 min; 95°C 1 min, 50°C 45 s, 72°C 1 min, 30 cycles; 72°C 10 min. The PCR product is sequenced (synbio-tech, Jiangshu, China), and the nucleotide sequence was blasted at the NCBI (https://blast.ncbi.nlm.nih.gov). The 16S rRNA sequence of *Lactobacillus salivarius* was deposited in GenBank (Genbank no. MT378407).

Assay of acid and bile tolerance of *Lactobacillus salivarius* were performed as previously described (12). Briefly, a suitable concentration of *Lactobacillus salivarius* were spread on normal MRS agar medium or MRS agar medium which were adjusted to pH = 3.0 or contained 0.3% bile salt. After incubation at 37°C for 2 h, the CFU per milliliter was assessed to determine acid/bile tolerance.

The antimicrobial activity of *Lactobacillus salivarius* was carried as described previously (12). *Escherichia coli* (CVCC1553), *Campylobacter jejuni* (CVCC3883), *Salmonella* Pullorum (CVCC1789), *Salmonella* Typhimurium (CVCC2220) and *Clostridium perfringens* (CVCC1125) were obtained from China veterinary culture collection center. 5 × 10^6^ indicator strain of *Campylobacter jejuni, Escherichia coli, Salmonella* Pullorum, *Salmonella* Typhimurium and *Clostridium perfringens* were grown on LB agar medium (Qingdao Hopebio Biology, Shandong, China), followed by embedding with sterilized Oxford cups into the LB agar plate. To each Oxford cup was added 150 μL of 10^7^ CFU *Lactobacillus* bacterial solution and the diameter of the inhibition area was determined after 24 h incubation at 37°C.

### Preparation of the Bacterial Strains

*Lactobacillus salivarius* was grown in MRS broth for 18 h under aerobic conditions at 37°C, and was added in the basal diet to a final concentration of 10^7^, 10^8^, and 10^9^ CFU/kg of feed. *Escherichia coli* O78 was aerobically cultured in LB broth (Hopebiol, Qingdao, China) for 18 h at 37°C, and orally administered to chickens at 10^6^ CFU *Escherichia coli* O78 on day 7 of age. The CFU was determined by counting the number of colonies grown on plates. Briefly, a gradient dilution series of *Lactobacillus salivarius* or *Escherichia coli* O78 were plated evenly on MRS or LB solid media in sterile plates. The plates were incubated at 37°C for 18-24 h and the colonies were counted based on the sample dilution.

### Growth Performance and Immune Organ Index

Weight and shank length were recorded individually at 4 and 8 weeks of age, the shank length was measured from the upper tibial joint to the third and fourth toes. Spleen, bursal and thymus gland tissues were sampled and weighed for each chicken at 4 and 8 weeks of age, the immune organ index (immune organ weight, mg/body weight, g) was measured.

### Fecal Indole and Skatole Content

Indole and skatole concentrations in the feces were detected by HPLC as previously described with some modifications (13). The standard substances of indole and skatole (Sigma-Aldrich, USA) were used to construct the standard curve. One grams of fecal sample was added to 4 mL methanol and mixed well, then water bath at 40°C for 20 min. The sample was placed at room temperature for 15 min and centrifuged at 12,000 g for 15 min. Sample size: 15 μL and the chromatographic column was a ZORBAX ECLIPSE XDB-C8 (5 μm, 4.6 × 150 mm) stainless steel column; column temperature: 30°C; fluorescence detector: excitation wavelength, 270 nm; emission wavelength, 350 nm; mobile phase: acetonitrile:water = 50:50; flow velocity: 1.0 mL/min.

### *Escherichia coli* O78 Detection in Ileal Contents

The bacterial DNA was extracted according to the manufacturer's guidelines (MagPure Stool DNA KF kit, Magen, China). Specific primers (synbio-tech, Jiangshu, China) used for PCR amplification, the primers used were as follows: *rfb*-F: CGATGTTG AGCGCAAGGTTG; *rfb*-R: TAGGTATTCCTGTTGCGGAG (14). The reaction system is 10 × PCR buffer 2.5 μL, forward and reverse primers 1 μL, DNA template 1 μL, TaqDNA polymerase 0.125 μL (TaKaRa, Dalian, China), and water added up to 25 μL. The product was connected to the PMD-19T vector and transferred to the *Escherichia coli* DH5α (Transgen, Beijing, China). The blue and white spots were screened and the suspected cloned colonies were cultured to extract the recombinant plasmid. The quantitative PCR reaction was carried out using a positive recombinant plasmid as a template. The reaction system was 10 μL PCR Mix (TaKaRa, Dalian, China) including the template DNA (1 μL), primers (1 μL), and the distilled water added up to 20 μL. PCR reaction conditions: 95°C 3 min; 95°C 15 s, 58°C 30 s, 72°C 35 s, 40 cycles. The absorption values of recombinant plasmid at 260 and 280 nm were determined by ultraviolet spectrophotometer (Bio-Rad, Hercules, CA, USA). The recombinant plasmid is diluted by a gradient of 10 times for the establishment of a standard curve. Plasmid copy = DNA mass concentration/DNA molecular weight; DNA molecular weight = DNA bases × 324.5; DNA mass concentration = 260 nm absorption value × 50 μg/mL × dilution multiple × 6.02 × 10^23^. Using the stander curve, *rfb* gene copies per gram of ileal contents was determined.

### Biochemical Analysis

Serum, heart and ileum tissues were collected. The creatine kinase (CK, A032-1-1), myocardial CK (CKMB, H197), lactic dehydrogenase (LDH, A020-2-2), malondialdehyde (MDA, A003-1-2), catalase (CAT), superoxide dismutase (SOD, A001-3-2), creatinine (CRE, C011-2-1), blood urea nitrogen (BUN, C013-2-1) alanine aminotransferase (ALT, C009-3-1) and aspartate aminotransferase (AST, C010-2-1) activities were measured using commercial kits according to the manufacturer's instructions (Jiancheng Institute of Bioengineering, Nanjing, China) and serum IFN-γ levels were detected by using a commercial kit (LS-F4229-1, Life Span Bio Sciences, USA). Ileal content was collected and mixed with 4 volume of PBS, and centrifuged at 1,200 g for 10 min. The supernatant used to secretory IgA (SIgA) detection by a commercial kit (H108) according to the manufacturer's instructions (Jiancheng Institute of Bioengineering, Nanjing, China).

### Quantitative Real-Time PCR (qRT-PCR) Analysis

The qRT-PCR was performed as described previously (15). Briefly, total RNA was extracted using TransZol Up Plus RNA Kit (Transgen, Beijing, China) following the manufacturer's instructions. 0.5 μg total RNA was reverse transcribed using the EasyScript® First-Strand cDNA Synthesis SuperMix (Transgen, Beijing, China). The mRNA expression levels were measured by TransStart® Tip Green qPCR SuperMix (Transgen, Beijing, China) on a Roche 480 real-time PCR system thermocycler. Each sample was analyzed in triplicates and target gene expression was analyzed by 2^−ΔΔ*Ct*^ method (16), following normalization with β-actin gene. The primers used are provided in Table 1.

**Table 1:** Primers used in qRT-PCR.

**Gene**	**Primer sequence (5^**′**^-3^**′**^)**	**References**
β-actin	F: GAGAAATTGTGCGTGACATCA R: CCTGAACCTCTCATTGCCA	(5)
Hsp70	F: TGTGTCCATCCTTACCATTGAG R: GCTTGTGCTTACCCTTGAACTC	(6)
Hsp90	F: TCAGACTTGATAACGGTGAACCT R: TGTCTTCTCCTCCTTCTCCTCTT	(6)
Occludin	F: TCGTGCTGTGCATCGCCATC R: CGCTGGTTCACCCCTCCGTA	(17)
ZO-1	F: GCGCCTCCCTATGAGGAGCA R: CAAATCGGGGTTGTGCCGGA	(17)
Claudin1	F: TGGAGGATGACCAGGTGAAGA R: CGAGCCACTCTGTTGCCATA	(18)
CRYAB	F: TCATGGGAAACACGAGGAGC R: ACACAGCAAACTTTCGTGGC	(19)
Hsp 27	F: ACACGAGGAGAAACAGGATGAG R: ACTGGATGGCTGGCTTGG	(20)

### Fecal Shorter-Chain Fatty Acids (SCFAs) Detection

The fecal SCFAs concentrations were detected as previously described (21) with some modifications. The standard substances of acetate, propionate and butyrate (Solarbio, Beijing, China) were used to construct the standard curve. Fecal samples were collected and mixed with 4 equal volume of PBS, and centrifuged at 12,000 g for 15 min. The internal standard was 2-ethylbutyric acid. The concentrations of acetate, propionate and butyrate in the fecal samples were determined using a gas chromatography (GC) system (7890B, Agilent). Nitrogen was supplied at a flow rate of 40 mL per min as a carrier gas. The initial oven temperature was set at 80°C for 0.5 min, then adjusts the temperature by rising 5°C per min for 10 min and held for 2 min, then increased 20°C per minute for 5 min and held for 1 min. The flow rates of hydrogen and air were 40 and 450 mL per min, respectively.

### Serum Specific Antibody Detection

Serum specific antibody was detected by a chicken infectious bursal disease virus antibody detection kit (IDEXX® Laboratory, Inc., United States) according to the manufacturer's instructions. The relative levels of antibody titer were detected by calculating the sample to positive (S/P) ratio as [(mean of sample optimal density)-(mean of negative control optimal density)]/[(mean of positive control optimal density)-(mean of negative control optimal density). Endpoint titer was calculated with the equation: Log 10 titer = 1.09 (Log 10 S/P) + 3.36. The presence of antibody was reported as positive when S/P ratio is > 0.2 and negative when S/P ratio is ≤ 0.2.

### Lymphocyte Proliferation Index Measurement

The chicken spleen samples were collected and the spleen cells were blown into the plate with 5 mL HBSS (Hank's Balanced Salt Solution). After mesh screen, cells were transferred into the centrifuge tube. Red blood cells were removed with 0.01 M Trimethyl-amino methane containing 0.83% ammonium chloride. The cells were washed with the full HBSS and the concentration of the cells in each group was adjusted to 5.0 × 10^5^ cells/mL. The 100 μL cells were seeded in 96-well plates and stimulated with 5 μg/mL Concanavalin (ConA, Solarbio, Beijing, China) and 8 μg/mL LPS (Sigma-Aldrich Inc, St. Louis, MO, USA), respectively. The plates were cultured at 37°C and 5% CO_2_ for 48 h. Cell proliferation was measured by MTT method. The calculation formula for the stimulation index (SI) is defined as follows: SI = (OD value of stimulation well – OD value of blank well)/(OD value of unstimulated well – OD value of blank well).

### Statistical Analysis

The data are expressed as mean ± SD. Statistical analyses were performed using GraphPad Prism 8.0 (GraphPad Software). Statistical significance were determined by one-way or two-way ANOVA with Tukey tests for multiple-group comparisons. The level of significance was set at *P* < 0.05.

## Results

### Dietary *Lactobacillus salivarius* Supplementation Improves Growth Performance

**Figure 1:**
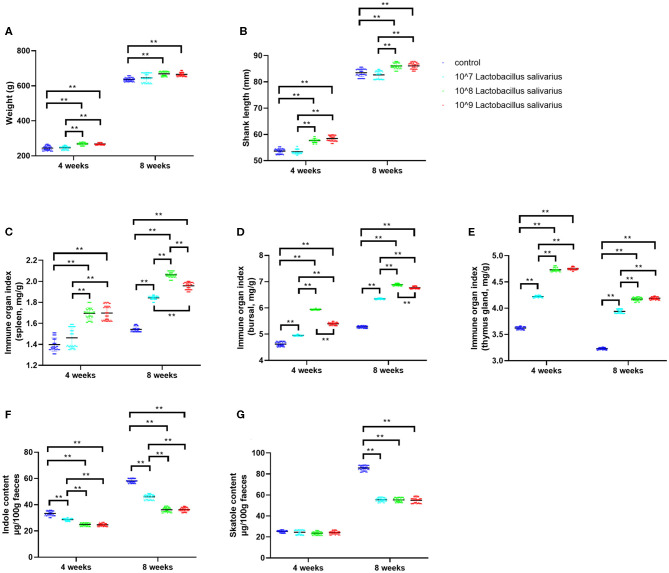
Effects of dietary *Lactobacillus salivarius* supplementation on growth performance of chickens. **(A,B)** Effects of dietary supplementation with *Lactobacillus salivarius* on body weight and shank length at 4 and 8 weeks of age, respectively (*n* = 60). **(C–E)** Immune organ indices of spleen, bursa and thymus, which were measured at 4 and 8 weeks of age (*n* = 60). **(F–G)** Indole and skatole concentrations were measured at 4 and 8 weeks of age (*n* = 60). Each point represents a single bird and bars represent mean ± SD. Data were analyzed by one-way ANOVA with Tukey tests. ** indicates *P* < 0.01.

In the present study, the selected *Lactobacillus salivarius* showed good performance to resist acid (pH = 3) and 0.3% bile salt (Supplementary Figure 1A). Furthermore, *Lactobacillus salivarius* showed good antimicrobial activities to five pathogenic bacteria, especially for *Escherichia coli* O78 (Supplementary Figure 1B). On this basis, we further evaluated the effects of *Lactobacillus salivarius* on the growth performance of chickens in the first 8 weeks. After 4 and 8 weeks of feeding, chickens fed with the diet of 10^8^ and 10^9^ CFU/kg *Lactobacillus salivarius* showed heavier weight and longer shank length (*P* < 0.01) than the control diet chickens (Figures 1A,B). However, there were no significant differences between chickens fed with the diet of 10^7^ CFU/kg *Lactobacillus salivarius* and the control group after 4 and 8 weeks (Figures 1A,B). The immune organ indices of the spleen and bursa were significantly increased (*P* < 0.01) in 10^8^ and 10^9^ CFU/kg *Lactobacillus salivarius* groups after 4 weeks, and the immune organ indices of the spleen and bursa were significantly increased (*P* < 0.01) in 10^7^, 10^8^, and 10^9^ CFU/kg *Lactobacillus salivarius* groups after 8 weeks (Figures 1C,D). The immune organ index of the thymus was significantly increased (*P* < 0.01) in 10^7^, 10^8^, and 10^9^ CFU/kg *Lactobacillus salivarius* groups after 4 and 8 weeks (Figure 1E). Chickens in 10^7^, 10^8^, and 10^9^ CFU/kg *Lactobacillus salivarius* groups showed significantly reduced (*P* < 0.01) indole content in feces after 4 and 8 weeks (Figure 1F), and the skatole content in feces were significantly decreased (*P* < 0.01) in 10^7^, 10^8^, and 10^9^ CFU/kg *Lactobacillus salivarius* groups after 8 weeks (Figure 1G). Chickens fed a diet with 10^9^ CFU/kg *Lactobacillus salivarius* showed higher growth performance than (*P* < 0.01) the control group, but showed no significant difference compared to 10^8^ CFU/kg *Lactobacillus salivarius* group (*P* > 0.05). Based on the above results, chickens fed with the diet of 10^8^ CFU/kg *Lactobacillus salivarius* were chosen for the following experiments.

### Effects of *Lactobacillus salivarius* on the Response of Chickens to *Escherichia coli* O78 Challenge

**Figure 2:**
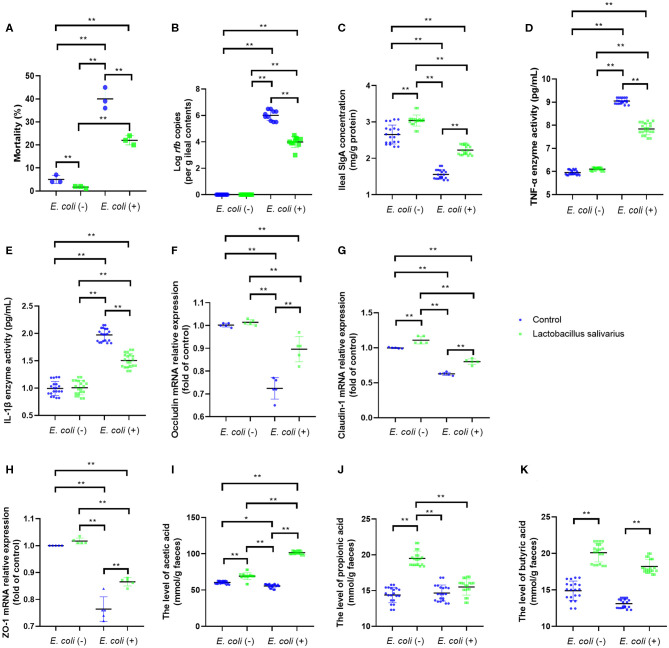
Effects of dietary *Lactobacillus salivarius* supplementation on the response of chickens to pathogenic *Escherichia coli* O78 challenge. **(A)** Mortality of the chickens following *Escherichia coli* O78 challenge, which was measured at 8 weeks of age (*n* = 3). Each point represents the result from an independent experiment and bars represent mean ± SD. **(B)** *Escherichia coli* O78 colonization in ileal contents at 7 days post-*Escherichia coli* infection (*n* = 10). **(C)** Secretory IgA (sIgA) concentration of ileum mucosa, which was measured 7 days post-*Escherichia coli* challenge (*n* = 20). **(D,E)** Cytokines of ileal tissues, which were measured 7 days post-*Escherichia coli* O78 challenge (*n* = 20). **(F–H)** Tight junction related gene mRNA expression of ileum, which were measured 7 days post-*Escherichia coli* challenge (*n* = 5). **(I–K)** Influence of *Lactobacillus salivarius* on shorter-chain fatty acids (SCFAs) levels of feces, which were measured 7 days post-*Escherichia coli* challenge (*n* = 20). ** indicates *P* < 0.01. Each point represents a single bird and bars represent mean ± SD **(B–K)**. Two-way ANOVA for repeated measurements, followed by Tukey tests. * indicates *P* < 0.05; ** indicates *P* < 0.01.

After 8 weeks of feeding, chickens fed with *Lactobacillus salivarius* showed lower mortality (*P* < 0.01) than the control diet chickens (Figure 2A). Compared with the control group, chickens fed with *Lactobacillus salivarius* significantly decreased mortality (*P* < 0.01) post-*Escherichia coli* O78 challenge (Figure 2A). Moreover, *Lactobacillus salivarius* supplementation significantly decreased (*P* < 0.01) *Escherichia coli* O78 colonization in digestive tract (Figure 2B). Intestinal SIgA reflects the intestinal immunity state, chickens fed with *Lactobacillus salivarius* exhibited markedly higher intestinal SIgA levels than the control group (Figure 2C, *P* < 0.05). Compared to control group, chickens fed with *Lactobacillus salivarius* significantly increased (*P* < 0.01) intestinal SIgA levels and decreased (*P* < 0.01) pro-inflammatory cytokines TNF-α and IL-1β levels post-*Escherichia coli* O78 challenge (Figures 2C–E). *Escherichia coli* O78 challenge significantly down-regulated the mRNA expression levels of ileal occludin, Claudin-1, and ZO-1 (Figures 2F–H, *P* < 0.01) compared to control group, which were reversed by *Lactobacillus salivarius* supplementation (Figures 2F–H, *P* < 0.01). Chickens fed with *Lactobacillus salivarius* showed increased levels (*P* < 0.01) of acetic acid, propionic acid and butyric acid in comparison with control group, and showed significantly higher levels (*P* < 0.01) of acetic acid and butyric acid post-*Escherichia coli* O78 challenge (Figures 2I–K).

### Effects of *Lactobacillus salivarius* on the Response of Chickens to Acute Heat Stress

**Figure 3:**
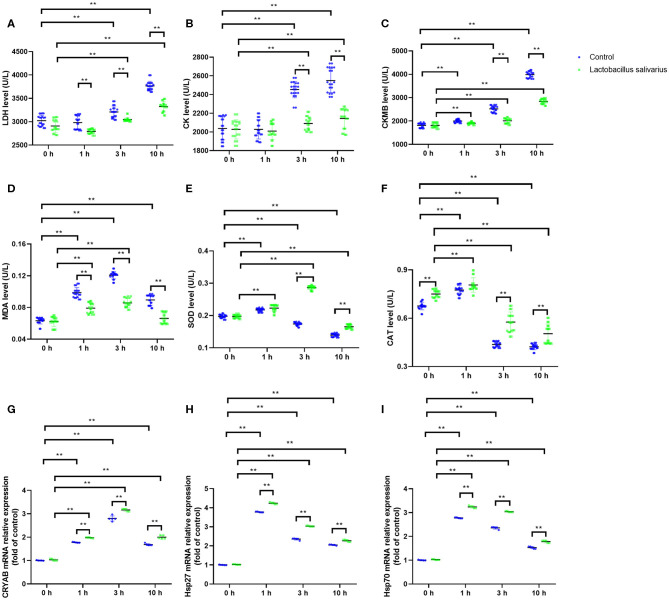
Effects of dietary *Lactobacillus salivarius* supplementation on the cardiac response of chickens to acute heat stress. **(A–C)** Cardiac damage-related enzymes of serum, which were measured 0, 1, 3, 10 h post-acute heat stress (*n* = 20). **(D–F)** Oxidative stress-related enzymes of heart tissue, which were measured 0, 1, 3, 10 h post-acute heat stress (*n* = 20). **(G–I)** CRYAB, Hsp27, and Hsp70 mRNA expression levels of heart tissue in each group, which were measured 0, 1, 3, 10 h post-acute heat stress (*n* = 5). Each point represents a single bird and bars represent mean ± SD. Two-way ANOVA for repeated measurements, followed by Tukey tests. ** indicates *P* < 0.01.

LDH, CK and CKMB levels in the serum were significantly increased (*P* < 0.01) in control group, especially at 3 and 10 h (Figures 3A–C) after exposure to heat stress, which indicated that heat stress caused heart damage. Compared to control group, the LDH, CK, and CKMB levels in the serum were significantly decreased (*P* < 0.05) in *Lactobacillus salivarius* supplementation group during heat stress (Figures 3A–C). The levels of MDA increased significantly from 1 to 10 h, especially at 1 and 3 h (*P* < 0.01); the activities of SOD and CAT were significantly increased 1 h (*P* < 0.01) post-heat stress and decreased significantly (*P* < 0.01) from 1 to 10 h (Figures 3D–F). Compared to control group, chickens fed with *Lactobacillus salivarius* exhibited higher SOD and CAT enzyme activities (*P* < 0.01), and showed lower MDA levels (*P* < 0.01) during heat stress (Figures 3D–F). The results also showed that the mRNA expression levels of CRYAB, Hsp27, and Hsp70 increased significantly (*P* < 0.01) during heat stress (Figures 3G–I). Compared to control group, the mRNA expression levels of CRYAB, Hsp27, and Hsp70 were significantly increased (*P* < 0.01) in *Lactobacillus salivarius* supplementation group during heat stress (Figures 3G–I).

### Effects of *Lactobacillus salivarius* on the Response of Chickens to Circular Heat Stress

**Figure 4:**
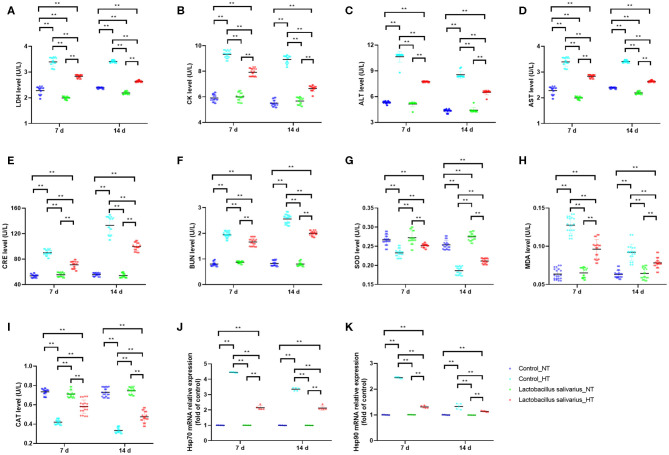
Effects of dietary *Lactobacillus salivarius* supplementation on the response of chickens to circular heat stress. **(A–F)** serum metabolic enzyme activity, which were measured at 7 and 14 days post-exposure to circular heat stress (*n* = 20). **(G–I)** Oxidative stress-related enzymes of ileum tissue, which were measured at 7 and 14 days after exposure to circular heat stress (*n* = 20). **(J–K)** Hsp70 and Hsp90 mRNA expression levels of ileum tissue in each group, which were measured 7- and 14-days post-exposure to circular heat stress (*n* = 5). Each point represents a single bird and bars represent mean ± SD. Two-way ANOVA for repeated measurements, followed by Tukey tests. ** indicates *P* < 0.01.

LDH, CK, ALT, AST, CRE, and BUN levels in the serum were significantly increased (*P* < 0.01) at 7 and 14 days post-circular heat stress exposure (Figures 4A–F), which indicated circular heat stress caused heart, liver and kidney damage. Compared to control group, the LDH, CK, ALT, AST, CRE, and BUN levels in the serum were significantly decreased (*P* < 0.01) in *Lactobacillus salivarius* supplementation group during heat stress (Figures 4A–F). The levels of MDA increased significantly (*P* < 0.01) and the activities of SOD and CAT were significantly decreased (*P* < 0.01) at 7 and 14 days post-circular heat stress exposure, which were alleviated by *Lactobacillus salivarius* supplementation (Figures 4G–I). Both the Hsp70 and Hsp90 mRNA expression levels (*P* < 0.01) of the control_HT group were significantly increased compared with the control_NT group at 7 and 14 days post-circular heat stress exposure (Figures 4J,K). Dietary supplementation with *Lactobacillus salivarius* significantly reduced (*P* < 0.01) the Hsp70 and Hsp90 mRNA expression levels on the 7th and 14th days of heat stress (Figures 4J,K).

### Effects of *Lactobacillus salivarius* on Immune Responses to Infectious Bursal Disease Virus (IBDV) Vaccine in Chickens of Oxidative Stress

**Figure 5:**
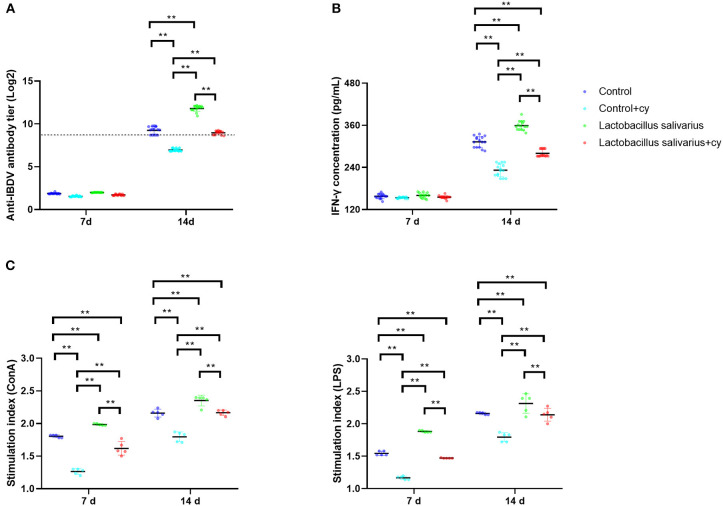
Effects of dietary *Lactobacillus salivarius* supplementation on immune response of chickens. **(A)** Serum anti-IBDV specific antibody titers in chickens, which were measured 7- and 14-days post-immunization (*n* = 15). Log 2 titers below 8.63 (which corresponds to S/P ratio <0.2) are considered negative and above 8.63 (S/P ratio > 0.2) are considered positive. **(B)** Levels of serum IFN-γ in chickens, which were measured 7- and 14-days post-immunization (*n* = 15). **(C)** Lymphocyte proliferation response in chickens, which were measured 7- and 14-days post-immunization (*n* = 5). Each point represents a single bird and bars represent mean ± SD. Two-way ANOVA for repeated measurements, followed by Tukey tests. ** indicated *P* < 0.01.

Compared to control group, there were significantly reduced levels (*P* < 0.01) of serum specific antibody levels of IBDV and serum IFN-γ in control + cy group post 14 days immunization; a significantly elevated levels (*P* < 0.01) of serum specific antibody levels of IBDV and serum IFN-γ were noted in *Lactobacillus salivarius* group post 14 days immunization (Figures 5A,B). Compared to control + cy group, *Lactobacillus salivarius* + cy group showed significantly increased (*P* < 0.01) serum specific antibody levels of IBDV and serum IFN-γ levels post 14 days immunization (Figures 5A,B). Compared to control group, cy significantly reduced lymphocyte proliferation (*P* < 0.01) induced by ConA and LPS post 7- and 14-days immunization; Compared to control group, *Lactobacillus salivarius* supplementation significantly enhanced (*P* < 0.01) lymphocyte proliferation induced by ConA and LPS post 7 and 14 days immunization (Figure 5C). Compared to control + cy group, *Lactobacillus salivarius* + cy group showed significantly enhanced (*P* < 0.01) lymphocyte proliferation induced by ConA and LPS post 7 and 14 days immunization (Figure 5C).

## Discussion

Probiotics are microorganisms that can enter the digestive tract alive and are beneficial to host health when ingested in sufficient quantities (21). Previous studies have confirmed the positive effects of *Lactobacillus salivarius* on growth performance and non-specific immunity levels of chickens (22, 23). For example, dietary supplementation of the *Lactobacillus salivarius* mixture improved body weight, body weight gain and feed conversion ratio, and improved intestinal histomorphology of broilers (22). *Lactobacillus salivarius* CTC2197 could prevent *Salmonella enteritidis* colonization as demonstrated by oral *Lactobacillus salivarius* CTC2197 together with *Salmonella enteritidis* and the *Salmonella enteritidis* was completely disappeared from chickens after 21 days (23). In the present study, multiple beneficial effects of *Lactobacillus salivarius* were studied in chickens, including growth performance, pathogenic bacterial intestinal colonization, immune responses and response to heat stress. It has been examined that *Lactobacillus salivarius* supplementation significantly improved the production performance-related indices of laying hens and also promoted immunological parameters, which is perhaps via inhibiting intestinal pathogens and thus reducing the nutrient consumption required for maintaining immunological activity (2). Odor emission from chicken excreta have adverse impacts on poultry production and surrounding environment (13). There has been clear evidence that odor compounds were mainly produced by microbial degradation of certain substrates in the intestine (13). Therefore, the production of odor compounds can be influenced by diet adjustment. The addition of *Lactobacillus* in the diet promoted the absorption of inorganic ions by intestinal epithelial cells, thus improved the absorption efficiency of nutrients, such as calcium and phosphorus, and increased the apparent nutrient digestibility of dry matter, nitrogen, and gross energy (24, 25). Similarly, the present study confirmed that *Lactobacillus salivarius* supplementation significantly reduced concentrations of fecal indole and skatole, which are the major odor-causing compounds.

*Escherichia coli* is widely found in nature, and exists in air, water and feed. Upon favorable conditions and suppression of immunity in chickens, *Escherichia coli* could multiply in a large number in the body and cause diseases, such as peritonitis, salpingitis, and pneumonitis (21). Good breeding management is the key to ensure the low incidence of the disease. For example, temperature, humidity, ventilation and fecal treatment are closely related to the occurrence of colibacillosis. In addition, proper feed additive prevention is also an indispensable measure to prevent the occurrence of *Escherichia coli* disease (5, 21). Previous study showed that dietary addition of *Lactobacillus plantarum* could improve the intestinal health and reduce the mortality of chickens suffering from *Escherichia coli* challenge (21). In this study, the *Lactobacillus salivarius* showed a protective role against *Escherichia coli* O78 challenge by decreasing *Escherichia coli* O78 colonization. SCFAs, mainly acetic acid, propionic acid and butyric acid, are mainly derived from the metabolism of intestinal microbiota, high levels of SCFAs inhibit the growth and reproduction of pathogenic microorganism (26). Previous study also indicated that *Lactobacillus* could enhance the intestine barrier by increasing the concentration of SCFAs (21).

Chickens are particularly vulnerable to high temperatures due to the lack of sweat glands on their feathers and skin (27). In addition to reduce the room temperature by spraying water, negative effects of heat stress can be alleviated by feeding anti-oxidative substances in poultry production (6, 27). Previous studies have evaluated the antioxidant activity of various *Lactobacillus* strains and indicated that *Lactobacillus salivarius* showed good anti-oxidative properties (28, 29). In the present study, *Lactobacillus salivarius* supplementation effectively alleviated the organ damage and enhanced anti-oxidative capacity in both acute heat stress and circular heat stress condition. Heat shock proteins (HSPs) are a class of protective proteins, activated during by high temperature and maintain normal physiological activities (6, 21). In the present study, the HSPs mRNA expression levels of heart tissues were significantly increased post-acute heat stress in control group and further increased HSPs mRNA expression levels were detected in *Lactobacillus salivarius* group, which indicated that *Lactobacillus salivarius* supplementation reduces the negative effects of acute heat stress by inducing HSPs expression. We have also noted that the control group exhibited relatively stable high levels of ileal HSPs expression when chickens were exposed to circular heat stress. However, the *Lactobacillus salivarius* group showed significantly reduced HSPs expression levels, this is probably because of chickens fed with *Lactobacillus salivarius* gradually adapted to heat stress and developed a certain degree of tolerance (6).

Cyclophosphamide (Cy) usually used to induce immunosuppression and oxidative stress in animal models (8). We furthermore assessed the effects of *Lactobacillus salivarius* supplementation on immune responses to infectious bursal disease vaccine in chickens with oxidative stress induced by Cy. Our results showed that *Lactobacillus salivarius* supplementation enhanced immune responses to infectious bursal disease vaccine in chickens with cy treatment. This may be due to *Lactobacillus salivarius* enhanced the body's ability to resist oxidative stress, which has been confirmed by previous studies (28, 29), and our results are consistent with these findings. In addition, we also found that *Lactobacillus salivarius* group exhibited an enhanced immune response after immunization compared to control group in chickens without cy treatment. This is consistent with previous results that *Lactobacillus* can be used as immune adjuvant to enhance antigen-specific immune responses (30, 31).

In conclusion, the present study demonstrated that the *Lactobacillus salivarius* supplementation improved growth performance, enhanced immune response and ameliorated the negative effects of stress response and *Escherichia coli*. These findings support *Lactobacillus salivarius* as an effective substitute for antibiotics in the poultry industry.

## Data Availability Statement

The 16S rRNA sequence of *Lactobacillus salivarius* was deposited in GenBank (Genbank No. MT378407).

## Ethics Statement

All animal experiments were conducted under the approval of Laboratory Animal Ethics Committee of Northeast Agricultural University (Heilongjiang, China) in accordance with Laboratory animal-Guideline for ethical review of animal welfare (GB/T35892-2018, National Standards of the People's Republic of China).

## Author Contributions

JW, CC, and JL designed the study and wrote the paper. JW, MI, and YG finished the experiments. MI made the critical revisions to the paper.

## Conflict of Interest

The authors declare that the research was conducted in the absence of any commercial or financial relationships that could be construed as a potential conflict of interest.
