# Supplementary material for: Assessment of Probiotic Properties of Lactobacillus salivarius Isolated From Chickens as Feed Additives
Source: Front Vet Sci. 2020 Jul 17;7:415. doi: 10.3389/fvets.2020.00415 (PMC7379216; doi:10.3389/fvets.2020.00415)
Supplement: Supplementary file 1 [file Data_Sheet_1.docx]

**
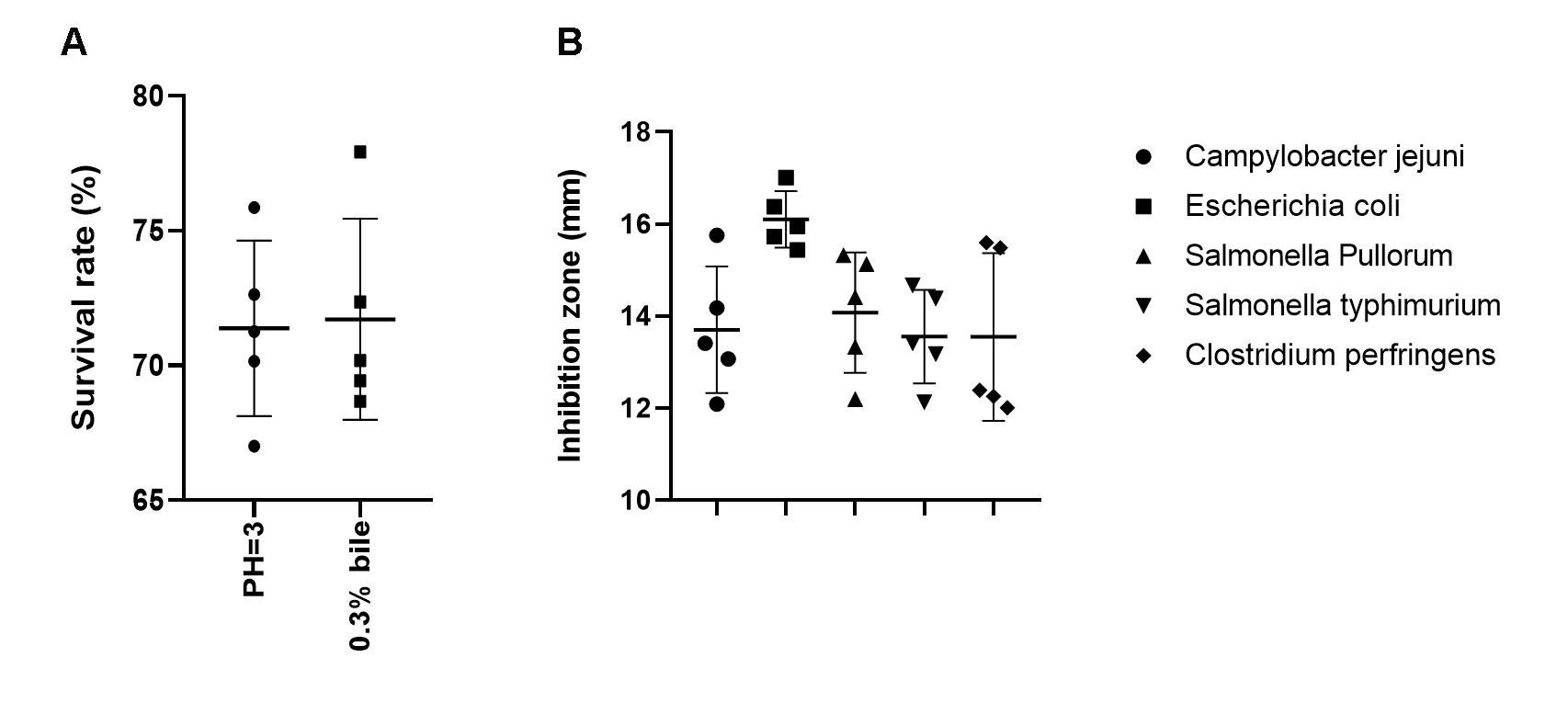
**

**Supplemental figure 1** Assessment of probiotic properties of *Lactobacillus salivarius*. **(A)** The survival rate of isolated *Lactobacillus salivarius*. The MRS medium adjusted to pH 3.0 to test acid tolerance and contained 0.3% bile salt to test bile tolerance for 2 h. Bars represent means ± SD of five independent experiments. **(B)** Antibacterial activity of *Lactobacillus salivarius* against five pathogenic bacteria (*Escherichia coli* O78, *Campylobacter jejuni*, *Salmonella* Pullorum, *Salmonella* Typhimurium and *Clostridium perfringens*. Bars represent means ± SD of five independent experiments.
